# [Retracted] miRNA-30a-3p inhibits metastasis and enhances radiosensitivity in esophageal carcinoma by targeting insulin-like growth factor 1 receptor

**DOI:** 10.3892/mmr.2026.13822

**Published:** 2026-02-09

**Authors:** Yanxin Fan, Xiuhua Bian, Pudong Qian, Jing Wen, Pengwei Yan, Yanhong Luo, Jing Wu, Qian Zhang

Mol Med Rep 20: 81–94, 2019; DOI: 10.3892/mmr.2019.10222

Following the publication of the above paper, it was drawn to the Editor's attention by a concerned reader that certain of the scratch-wound assay data within Fig. 10A, and the Transwell assay data shown in Figs. 2C and 10B, contained duplicated data panels within the figure parts, and also comparing between the figures in the case of Figs. 2C and 10B; moreover, Transwell assay data shown in Fig. 3B were strikingly similar to data that had already been submitted for publication to the journal *International Journal of Molecular Medicine* in a paper written by different authors at different research institutes.

In view of the fact that the abovementioned data had already apparently been submitted for publication prior to its submission to *Molecular Medicine Reports*, the Editor has decided that this paper should be retracted from the Journal. The authors were asked for an explanation to account for these concerns, but the Editorial Office did not receive a reply. The Editor apologizes to the readership for any inconvenience caused.

